# A Refined Carbohydrate-Rich Diet Reduces Vascular Reactivity Through Endothelial Oxidative Stress and Increased Nitric Oxide: The Involvement of Inducible Nitric Oxide Synthase

**DOI:** 10.3390/nu17152395

**Published:** 2025-07-22

**Authors:** Karoline Neumann, Nina Bruna de Souza Mawandji, Ingridy Reinholz Grafites Schereider, Emanuelle Coutinho de Oliveira, Julia Martins Vieira, Andressa Bolsoni-Lopes, Jones Bernardes Graceli, Julia Antonietta Dantas, Lorena Silveira Cardoso, Dalton Valentim Vassallo, Karolini Zuqui Nunes

**Affiliations:** 1 Postgraduate Program in Nutrition and Health, Health Sciences Center, Federal University of Espírito Santo, Vitória 29040-091, ES, Brazil; karolineneumanngomes@hotmail.com (K.N.); andressa.lopes@ufes.br (A.B.-L.); 2 Postgraduate Program in Physiological Sciences, Health Sciences Center, Federal University of Espírito Santo, Vitória 29040-091, ES, Brazil; ninamawandji@gmail.com (N.B.d.S.M.); ingridy_grafit@hotmail.com (I.R.G.S.); jbgraceli@gmail.com (J.B.G.); julia_antonietta@yahoo.com.br (J.A.D.); daltonv2@outlook.com (D.V.V.); 3 Undergraduate Program in Nursing and Obstetrics, Health Sciences Center, Federal University of Espírito Santo, Vitória 29040-091, ES, Brazil; emanuelle.c.oliveira@edu.ufes.br; 4 Undergraduate Program in Pharmacy, Health Sciences Center, Federal University of Espírito Santo, Vitória 29040-091, ES, Brazil; julia.vieira509@gmail.com; 5 Animal Science, College of Agricultural Sciences, Southern Illinois University, Carbondale, IL 62901, USA; 6 Integrated Colleges of Espírito Santo–FAESA, Cariacica 29145-000, ES, Brazil; lo-silveira@hotmail.com

**Keywords:** refined carbohydrate-rich diet, aorta, vascular reactivity, inflammation, oxidative stress

## Abstract

Background/Objectives: The consumption of refined carbohydrates has increased globally. It is associated with inflammation and oxidative stress, both recognized as risk factors for cardiovascular disease. This study investigated the effects of a refined carbohydrate-rich diet on the vascular reactivity of rat aorta. Methods: We acclimatized adult male Wistar rats for two weeks and then randomly assigned them to two experimental groups: a control (CT) group and a high-carbohydrate diet (HCD) group. The CT group received standard laboratory chow for 15 days, while the HCD group received a diet composed of 45% sweetened condensed milk, 10% refined sugar, and 45% standard chow. After the dietary exposure period, we evaluated the vascular reactivity of aortic rings, gene expression related to inflammation, superoxide dismutase activity, and biochemical parameters, including cholesterol, triglycerides, fasting glucose, and glucose and insulin tolerance. Results: The results demonstrate a reduction in vascular reactivity caused by endothelial alterations, including increased NO production, which was observed as higher vasoconstriction in the presence of L-NAME and aminoguanidine and upregulation of iNOS gene expression. In addition, increased production of free radicals, such as O_2_^-^, was observed, as well as immune markers like MCP-1 and CD86 in the HCD group. Additionally, the HCD group showed an increase in the TyG index, suggesting early metabolic impairment. GTT and ITT results revealed higher glycemic levels, indicating early signs of insulin resistance. Conclusions: These findings indicate that short-term consumption of a refined carbohydrate-rich diet may trigger oxidative stress and endothelial dysfunction, thereby increasing the risk of cardiovascular complications.

## 1. Introduction

The consumption of refined carbohydrates has been increasing worldwide, driven by globalization and greater access to ultra-processed foods [1,2,3]. This dietary pattern is characterized by a high intake of rapidly absorbed carbohydrates and low dietary fiber content. Such diets have been associated with the rising incidence of noncommunicable chronic diseases, particularly cardiovascular diseases (CVDs), which are considered the leading causes of death globally [4,5,6,7]. Population-based studies indicate that high carbohydrate intake is significantly associated with an increased risk of CVD and mortality [8,9,10,11,12,13].

In this context, the World Health Organization (WHO) recognizes refined carbohydrates as modifiable risk factors for cardiovascular diseases (CVDs), recommending that their intake be reduced to less than 10% of total energy consumption [14]. Recent studies have highlighted a significant increase in the consumption of refined carbohydrate-rich products, both among children—particularly during critical stages of development—and adults [3]).

Excessive intake of these carbohydrates has been shown to promote a range of metabolic and inflammatory alterations, such as increased visceral adiposity, glucose intolerance, hyperlipidemia, and elevated levels of inflammatory cytokines [15,16,17,18]. These changes have deleterious effects on various metabolic structures, including adipose tissue, pancreas, liver, and skeletal muscle, which may predispose individuals to the development of conditions such as obesity and CVDs [17].

Animal model studies have shown that refined carbohydrate-rich diets are associated with heightened inflammatory responses and oxidative stress [15,16,17,18,19,20], impairing the body’s anti-inflammatory defenses and directly contributing to significant cardiac changes such as myocardial inflammation, fibrosis, and contractile dysfunction [20]. Inflammatory changes and oxidative stress are recognized risk factors for endothelial dysfunction, which in turn leads to vascular dysfunction, playing a critical role in the development of vascular diseases [21].

Despite these findings, the precise mechanisms through which excessive refined carbohydrate consumption affects vascular and endothelial function remain unclear. This knowledge gap underscores the need for experimental studies to further explore these interactions. In this context, the present study aims to investigate the effects of a refined carbohydrate-rich diet on the vascular reactivity of aortic rings in male rats while also assessing short-term inflammation.

## 2. Materials and Methods

### 2.1. Animals

Adult male Wistar rats (8–10 weeks old, ±280 g) were housed in a temperature-controlled environment (23–25 °C), maintained on a 12 h light–dark cycle, and given standard rat chow or a high-carbohydrate diet (along with filtered water) ad libitum. This study was approved by the Ethics Committee on Animal Use of the Federal University of Espírito Santo (CEUA–UFES, protocol no. 16/2022) and conducted in accordance with the guidelines of the National Council for the Control of Animal Experimentation (CONCEA). This study did not employ formal blinding procedures during the experiments. However, animals were randomized into experimental groups based on similar age and body weight prior to treatment initiation. During the experiments, researchers were aware of the animals’ group allocation (control or HCD), which was necessary to prevent identification errors and ensure proper handling of the samples. It is important to emphasize that all procedures were conducted with the same methodological rigor and standardization across groups to minimize any bias in conduct or interpretation.

### 2.2. Experimental Groups

The animals were acclimatized for two weeks before being randomly assigned to two experimental groups: the control (CT) group and the high-carbohydrate diet (HCD) group. The CT group received standard laboratory chow (Nuvilab^®^—Curitiba, Paraná, Brazil) while the HCD group received a diet composed of 45% sweetened condensed milk, 10% refined sugar, and 45% standard chow. The macronutrient composition of the standard diet (4.0 kcal/g) included 65.8% carbohydrates, 3.1% fats, and 31.1% proteins. In comparison, the high-carbohydrate diet (4.4 kcal/g) consisted of 74.2% carbohydrates, 5.8% fats, and 20% proteins. It is worth noting that a high-carbohydrate diet contains at least 30% organic sugars, primarily sucrose [16,17]. A total of 70 animals were used, with 35 rats in the control group and 35 rats in the high-carbohydrate diet (HCD) group. Point variations in the final number of animals per experiment resulted from pre-established exclusion criteria, such as sample loss during processing, adverse events unrelated to the protocol, and others.

### 2.3. Glucose and Insulin Tolerance Tests

On the twelfth day of treatment, the animals were subjected to a 6 h fast prior to intraperitoneal injection of glucose (2 g/kg body weight—20% glucose solution) or insulin (0.75 mU/g body weight of regular human insulin—Humulin, Lilly, Brazil). For the glucose tolerance test (GTT), blood was collected from the tail 0, 15, 30, 60, and 90 min after glucose administration. For the insulin tolerance test (ITT), blood was collected at 0, 3, 6, 9, 12, and 15 min after insulin administration. A glucometer (Accu-Chek Active, Roche, Brazil) was used to measure blood glucose levels in both tests. The interpretation of the GTT and ITT was based on the variation in blood glucose levels at each time point. The results were analyzed by comparing glucose concentrations at each time using Student’s *t*-test [22].

### 2.4. Body Parameters, Tissue Collection, and Glycemic Profile

We calculated body weight gain as the difference between the final and initial body weights (in grams) over the experimental period. Feed and water intake were measured as the difference between the amount provided and the amount remaining, with results expressed in g/day/animal. Feed efficiency was also calculated as the ratio of body mass gain (in grams) to energy intake (in kcal).

After 15 days of treatment, the animals underwent a 6 h fasting period, and fasting capillary blood glucose levels were measured prior to anesthesia. We collected blood samples from the tail artery and measured blood glucose using a glucometer (Accu-Chek Active, Roche, Brazil). The animals were then anesthetized with a combination of ketamine and xylazine (75 mg/kg and 10 mg/kg intraperitoneally). Anesthetic effectiveness was evaluated by the response to nociceptive stimuli, such as tail pinching, and additional doses were administered as needed.

Blood samples were also collected from the abdominal aorta, centrifuged to obtain serum, and stored at −20 °C for future analyses, including lipid profile and superoxide dismutase (SOD) enzyme activity. We collected and weighed the following adipose tissues: epididymal, mesenteric, subcutaneous, perirenal, retroperitoneal, and thoracic. All fat weights were normalized to the animals’ final body weight.

### 2.5. Lipid Profile Assessment

Total cholesterol, High-Density Lipoprotein (HDL), and triglycerides were quantified in serum samples using enzymatic colorimetric methods with commercial kits from Labtest Diagnóstica (Lagoa Santa, MG, Brazil; 202202 IDO01). All assays were performed according to the manufacturer’s instructions regarding standards and reagents. Quantifications were conducted using an ELISA reader and a Thermo Scientific Multiskan FC microplate photometer (Waltham, MA, USA). The triglyceride and glucose (TyG) index was also evaluated as previously reported (22, 23). Briefly, the TyG index was calculated using the following formula: fasting triglyceride (mg/dL) × fasting plasma glucose (mg/dL) levels [21,22,23].

### 2.6. Vascular Reactivity

The thoracic aorta was carefully dissected with and without the presence of perivascular adipose tissue (PVAT) and sectioned into 2–4 mm segments for reactivity experiments. For isometric tension recordings, each aortic ring was mounted in a 5 mL organ bath containing Krebs–Henseleit solution (KHS, in mM: 115 NaCl, 25 NaHCO_3_, 4.7 KCl, 1.2 MgSO_4_·7H_2_O, 2.5 CaCl_2_, 1.2 KH_2_PO_4_, 11.0 glucose, and 0.01 Na_2_EDTA), maintained at 37 °C and continuously aerated with a carbogenic gas mixture (95% O_2_–5% CO_2_), then stabilized for 30 min at a resting tension of 1 g [24].

Initially, aortic rings were exposed twice to 75 mM KCl. The first exposure assessed vessel functionality, while the second measured maximal tension development. Endothelial integrity was confirmed by the ability of phenylephrine (10^−3^ M) to induce contraction and acetylcholine (10^−2^ M) to induce relaxation. After three washes and stabilization of the baseline tone, a concentration–response curve to phenylephrine (10^−9^ M to 10^−4^ M) was performed to evaluate contractile function [24].

We carried out the same protocol in endothelium-denuded vessels. The endothelium was removed mechanically using a metal rod inserted into the vessel lumen and gently rubbed against the intimal surface to induce endothelial injury. Following stabilization, the preparations were precontracted with phenylephrine, and successful endothelium removal was confirmed by the inability of acetylcholine to induce relaxation (<10%) [24].

Subsequently, experimental protocols were conducted to investigate the pathways involved in vascular contractile function.

These experiments were performed by incubating the aortic rings in the organ bath with the following pharmacological agents for 30 min prior to phenylephrine stimulation in vessels with intact endothelium: a nonselective nitric oxide synthase (NOS) inhibitor (NG-nitro-L-arginine methyl ester, L-NAME, 100 mM), a selective inducible nitric oxide synthase (iNOS) inhibitor (aminoguanidine, 50 µM), a reactive oxygen species (ROS) scavenger (Tiron, 1 mM), a nonselective K^+^ channel blocker (tetraethylammonium, TEA, 2 mM), a superoxide dismutase (SOD) inhibitor (diethyldithiocarbamic acid, DETCA, 0.5 mM), and a hydrogen peroxide scavenger (catalase, 1000 U/mL) [24].

### 2.7. RNA Extraction and Real-Time PCR 

Total RNA was extracted from the thoracic aorta using Trizol reagent (Invitrogen Life Technologies, Waltham, MA, USA). RNA purity and concentration were assessed based on absorbance ratios at 260/280 and 260/230 nm using a NanoDrop spectrophotometer (Thermo Scientific, Waltham, MA, USA). Reverse transcription into complementary DNA (cDNA) was performed using the Superscript III cDNA kit (Thermo Scientific, Waltham, MA, USA). Gene expression was analyzed by quantitative real-time polymerase chain reaction (qPCR) using a QuantStudio™ system (Thermo Scientific) with SYBR Green fluorescent dye, as previously described [25]. Real-time PCR data were analyzed using the 2^−^ΔΔCT method, and results were expressed as the ratio of target gene expression to housekeeping gene expression (β-actin).

The following genes were evaluated: inducible nitric oxide synthase (iNOS), monocyte chemoattractant protein-1 (MCP-1), and cluster of differentiation 86 (CD86). Primer sequences were as follows: β-actin (Forward: 5′ ACACCCGCCACCAGTTCG 3′; Reverse: 5′ CCCACGATGGAGGGGAAGAC 3′); CD86 (Forward: 5′ AAGACATGTGTAACCTGCACCA 3′, Reverse: 5′ AAGCTTGCCCTCTTCACAGGA 3′); MCP-1 (Forward: 5′ TGTCTCAGCCAGATGCAGTT 3′, Reverse: 5′ CAGCCGACTCATTGGGATCA 3′); iNOS (Forward: 5′ GGTGAGGGGACTGGACTTTT 3′; Reverse: 5′ TTCTCCGTGGGGATCA 3′); eNOS (Forward: 5′ GGCTGAGTACCCAAGCTGAG 3′; Reverse: 5′ ATTGTGGCTCGGGTGGATTT3′).

### 2.8. Superoxide Dismutase (SOD) Activity 

SOD activity was measured using a method based on the enzyme’s ability to inhibit the auto-oxidation of adrenaline. When adrenaline is added to an alkaline medium, it reacts with the superoxide anion to form adrenochrome, which has a peak absorbance at 480 nm. Therefore, lower absorbance of adrenochrome indicates greater SOD activity, as it indirectly reflects the enzyme’s ability to dismutate the superoxide anion and inhibit auto-oxidation.

For this assay, each well received 185 µL of glycine buffer, 5 µL of sample, 5 µL of catalase, and 5 µL of adrenaline. Absorbance was recorded immediately and read at 405 nm for 17 min, with measurements taken every 60 s [26].

### 2.9. Drugs and Reagents

L-phenylephrine hydrochloride, potassium chloride, L-NAME, acetylcholine chloride, Tiron, catalase, TEA, aminoguanidine, and DETCA were purchased from Sigma-Aldrich (St. Louis, MO, USA). All salts and reagents used were of analytical grade and obtained from Sigma-Aldrich or Merck (Darmstadt, Germany). All drugs were diluted in distilled water.

### 2.10. Statistical Analysis

Results are expressed as mean ± standard error of the mean (SEM). One-way or two-way analysis of variance (ANOVA) was used to compare group means, followed by Bonferroni’s post hoc test when appropriate. Student’s *t*-test was used for comparisons between two means. We analyzed differences in the area under the curve (ΔAUC) between groups to compare the magnitude of treatment effects on contractile vascular responses. The area under the curve (AUC) was calculated using GraphPad Prism software (version 8.4.2; GraphPad Software, Inc., San Diego, CA, USA), and differences are presented as the percentage of ΔAUC between control and experimental groups. Values were considered statistically significant when *p* < 0.05.

## 3. Results

### 3.1. Effects of Refined Carbohydrate-Rich Diet Consumption on Body Parameters

The consumption of a refined carbohydrate-rich diet for 15 days did not result in greater body mass gain compared to the CT group; however, there was a reduction in both food and water intake (−25.92% and −41%, respectively), as well as an increase in feed efficiency (+20%) when compared to the CT group. Data related to these variables are detailed in Table 1.

The refined carbohydrate-rich diet did not lead to significant changes in adiposity, as presented in Table 2.

### 3.2. Effects of Refined Carbohydrate-Rich Diet Consumption on Glycemic and Lipid Profiles

Regarding the glycemic and lipid profile assessment, animals in the HCD group showed increased fasting capillary glucose levels and elevated serum triglyceride levels. However, no differences were observed in total cholesterol levels compared to the CT group. Furthermore, the analysis of the TyG index revealed a significant increase in the values observed in the HCD group compared to the CT group, as presented in Table 3.

### 3.3. Effects of Refined Carbohydrate-Rich Diet Consumption on Glucose Metabolism

**Figure 1:**
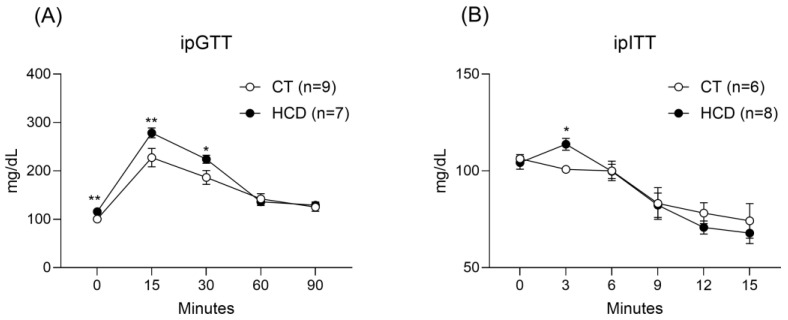
Evaluation of glucose metabolism. GTT (**A**) and ITT (**B**) in Wistar rats treated for 15 days with a control diet (CT) or a refined carbohydrate-rich diet (HCD). Data are expressed as mean ± SEM. * *p* < 0.05 vs. CT. ** *p* < 0.01 vs. CT. Student’s *t*-test was used. The number of animals is indicated in parentheses.

In the GTT, it was observed that animals in the HCD group exhibited higher blood glucose at time 0—prior to the intraperitoneal glucose solution infusion—and 15 min after the intraperitoneal glucose infusion compared with the CT group. During the ITT, an increase in glucose levels at 3 min was noted in the HCD group relative to the CT group, as shown in Figure 1.

### 3.4. Effects of Refined Carbohydrate-Rich Diet Consumption on Aortic Vascular Reactivity

To assess vascular reactivity, we initially exposed aortic rings to KCl in the presence and absence of PVAT. Exposure to KCl elicited a similar contractile response in both groups, regardless of PVAT presence, suggesting preserved vascular smooth muscle integrity (CT: 2.3 ± 0.40 g; *n* = 10 and HCD: 2.3 ± 0.29 g; *n* = 10; PVAT CT: 2.54 ± 0.54 g; *n* = 11 and PVAT HCD: 2.46 ± 0.46 g; *n* = 11).

**Figure 2:**
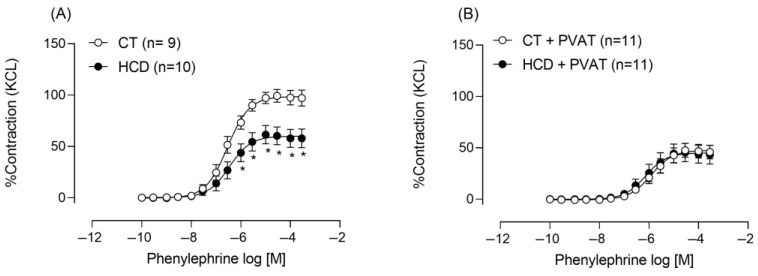
Concentration–response curves to phenylephrine (Phe) in thoracic aorta rings with (**A**) and without (**B**) the presence of PVAT. Each point represents the mean ± SEM. * *p* < 0.05, HCD vs. CT. The 95% CI = (CT: 93.27 to 104.6; HCD: 53.58 to 67.85). Two-way ANOVA followed by Bonferroni post hoc test. The number of animals is indicated in parentheses.

Dietary exposure did not alter the resting tension of the rings or the contractile response to phenylephrine in the presence of PVAT. However, without PVAT, phenylephrine-induced contractile responses were reduced in the HCD group (Figure 2).

**Figure 3:**
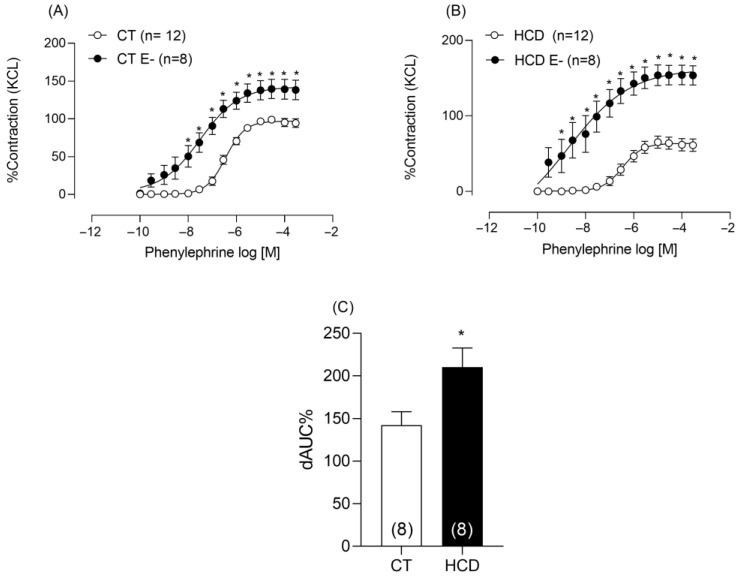
Effects of endothelium removal (E–) on Phe-induced vasoconstriction in aortic rings from CT (**A**) and HCD (**B**) groups. Each point represents the mean ± SEM. * *p* < 0.05, HCD vs. CT. The 95% CI = (CT:92.92 to 101.1; CT É-: 129.8 to 165.7; HCD: 57.7 to 70.0; HCD É-: Two-way ANOVA followed by Bonferroni post hoc test. (**C**) Percentage difference in the area under the concentration–response curve to phenylephrine (ΔAUC) for comparison between groups. Student’s *t*-test was used. * *p* < 0.05, HCD vs. CT. The number of animals is indicated in parentheses.

Given that altered vascular reactivity was observed in the absence of PVAT, subsequent experiments were conducted without this tissue, focusing on endothelial mechanisms. The endothelium was mechanically removed to explore whether the reduction in reactivity was related to endothelial factors. Phenylephrine reactivity increased in both groups (Figure 3A,B), but this increase was greater in the HCD group, as shown by the ΔAUC (Figure 3C).

**Figure 4:**
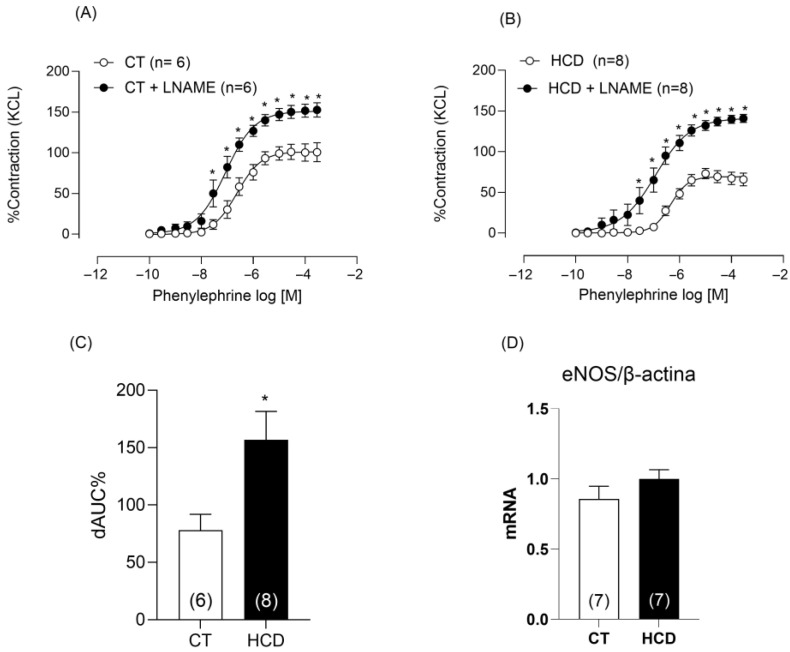
Effects of L-NAME on Phe-induced vasoconstriction. Effects of L-NAME (100 µM) on vasoconstriction in aortic rings from CT (**A**) and HCD (**B**) groups. Each point represents the mean ± SEM. * *p* < 0.05. HCD vs. CT. The 95% CI (CT: 93.57 to 110.0; CT + LNAME: 142.5 to 161.0; HCD: 64.48 to 74.29; HCD + LNAME: 130.5 to 165.7). Two-way ANOVA followed by Bonferroni post-test. (**C**) Percentage difference in the area under the concentration–response curve to phenylephrine (ΔAUC) for comparison between groups. (**D**) Messenger RNA concentration of *eNOS*. Student’s *t*-test was used on ΔAUC%. * *p* < 0.05, HCD vs. CT. The number of animals is indicated in parentheses.

Considering NO as the primary vasodilator produced by the endothelium, aortic rings were incubated with the NOS inhibitor L-NAME. L-NAME incubation increased reactivity in both groups (Figure 4A,B), with a more pronounced effect in the HCD group, as indicated by the area under the curve (Figure 4C). Since L-NAME is not specific to the different NOS isoforms, we evaluated the gene expression of eNOS and found no difference between the groups (Figure 4D).

**Figure 5:**
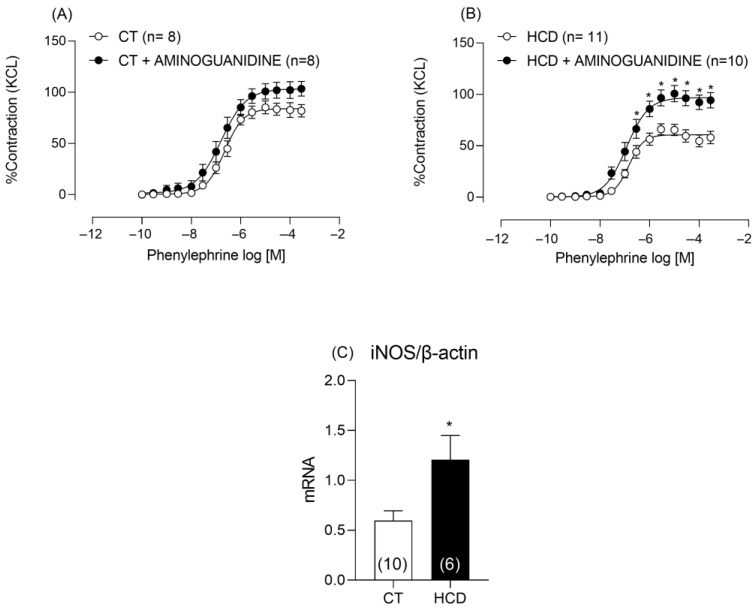
Effects of aminoguanidine (50 µM) on Phe-induced vasoconstriction and messenger RNA concentration of *iNOS*. Effects of aminoguanidine (50 Μm) on vasoconstriction in aortic rings from CT (**A**) and HCD (**B**) groups. Each point represents the mean ± SEM. * *p* < 0.05. HCD vs. CT. The 95% CI = (CT: 79.56 to 88.72; CT + AMINOGUANIDE: 96.32 to 111.4; HCD: 56.93 to 64.39; HCD + AMINOGUANIDINE: 91.12 to 102.9). Two-way ANOVA followed by Bonferroni post-test. (**C**) Messenger RNA concentration of *iNOS*. Data are expressed as mean ± SEM. * *p* < 0.05 HCD vs. CT. Student’s *t*-test was used. The number of animals used is indicated in parentheses.

Given the increased NO levels, the involvement of iNOS was investigated. Incubation with aminoguanidine only increased the contractile response in aortic rings from the HCD group (Figure 5A,B), confirming the hypothesis that iNOS contributed to increased NO production. Additionally, gene expression analysis revealed an upregulation of iNOS mRNA (Figure 5C).

**Figure 6:**
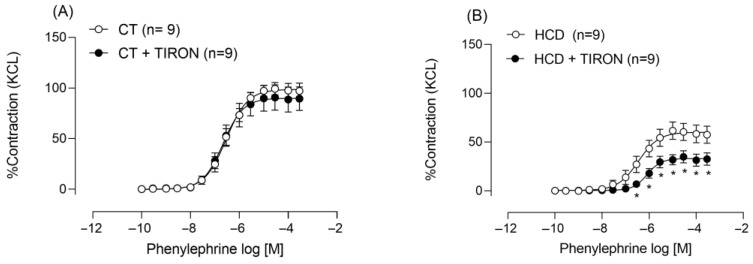
Effects of Tiron on Phe-induced vasoconstriction. Effects of Tiron (1 mM) on vasoconstriction in aortic rings from CT (**A**) and HCD (**B**) groups. Each point represents the mean ± SEM. * *p* < 0.05. HCD vs. CT. The 95% CI = (CT: 93.27 to 104.6; CT + TIRON: 81.71 to 99.4; HCD: 53.6 to 67.93; HCD + TIRON: 29.28 to 37.23). Two-way ANOVA followed by Bonferroni post-test. The number of animals used is indicated in parentheses.

To investigate the contribution of reactive oxygen species, we incubated aortic rings with Tiron, a free radical scavenger. Tiron only reduced phenylephrine-induced contraction in the HCD group (Figure 6A,B), suggesting the presence of vasoconstrictor free radicals exclusively in this group.

**Figure 7:**
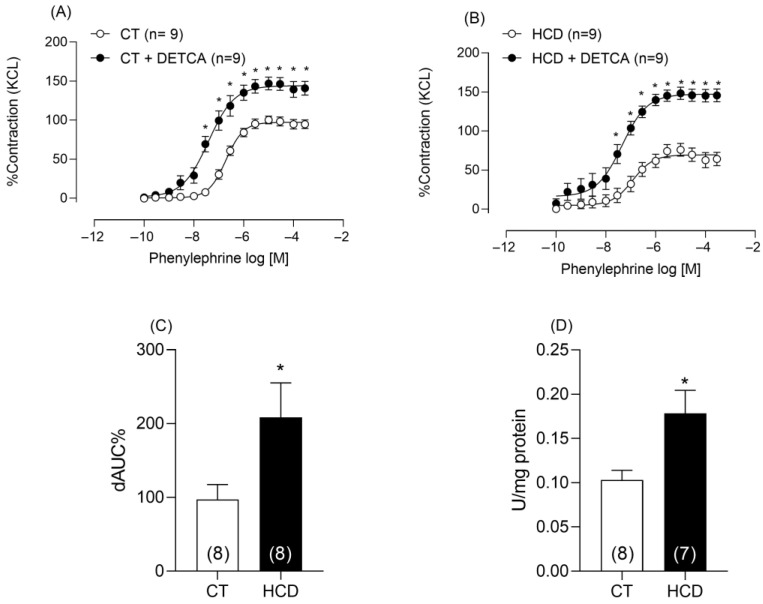
Effects of DETCA on Phe-induced vasoconstriction and SOD activity. Effects of DETCA (0.5µM) on vasoconstriction in aortic rings from CT (**A**) and HCD (**B**) groups. (**D**) SOD activity. Each point represents the mean ± SEM. * *p* < 0.05. HCD vs. CT. Two-way ANOVA followed by Bonferroni post-test. (**C**) Percentage difference in the area under the concentration–response curve to phenylephrine (ΔAUC) for comparison between groups. (**D**) SOD activity. *T*-test was used on ΔAUC% * *p* < 0.05. HCD vs. CT. The number of animals used is indicated in parentheses.

Considering the increase in free radicals, this study examined whether the consumption of a refined carbohydrate-rich diet altered the function of the antioxidant enzyme superoxide dismutase. To this end, rings were incubated with DETCA, an SOD inhibitor. DETCA increased phenylephrine-induced contraction in both groups (Figure 7A,B); however, this increase was greater in the HCD group, as shown by the area under the curve (Figure 7C), suggesting greater SOD involvement in this group. Additionally, SOD activity was increased in the HCD group (Figure 7D).

**Figure 8:**
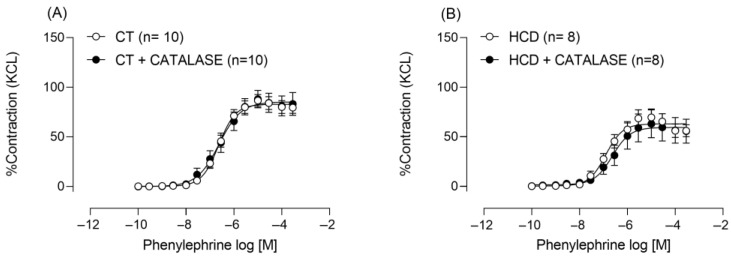
Effects of catalase on Phe-induced vasoconstriction. Effects of catalase (1000 U/mL^−1^) on vasoconstriction in aortic rings from CT (**A**) and HCD (**B**) groups. Each point represents the mean ± SEM. HCD vs. CT. Two-way ANOVA followed by Bonferroni post-test. The number of animals used is indicated in parentheses.

The observed increase in SOD activity may lead to greater production of hydrogen peroxide (H_2_O_2_), a reactive oxygen species that, depending on its concentration, can exert vasodilatory effects [24,27]. To investigate this possibility, we incubated aortic rings with catalase. However, catalase incubation did not affect contractile responses in either group (Figure 8), suggesting that although H_2_O_2_ may play a role in vascular reactivity, its concentration was insufficient to induce a significant effect under the experimental conditions. This result indicates that additional factors beyond H_2_O_2_ may have influenced vascular reactivity in the experimental groups or that alternative signaling pathways may have counteracted the potential vasodilatory effect of H_2_O_2_.

**Figure 9:**
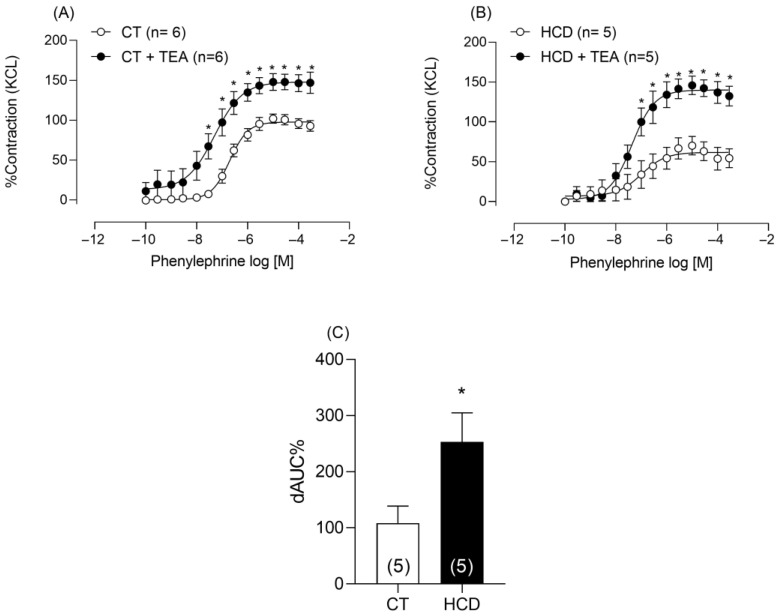
Effects of TEA on Phe-induced vasoconstriction. Effects of the nonselective K^+^ channel blocker, TEA (2 mM), on vasoconstriction in aortic rings from CT (**A**) and HCD (**B**) groups. Each point represents the mean ± SEM. * *p* < 0.05. HCD vs. CT. Two-way ANOVA followed by Bonferroni post-test. (**C**) Percentage difference in the area under the concentration–response curve to phenylephrine (ΔAUC) for comparison between groups. *T*-test was used on ΔAUC% * *p* < 0.05. HCD vs. CT. The number of animals used is indicated in parentheses.

Potassium channels play a key role in NO-mediated vascular relaxation and are essential for regulating smooth muscle responses to phenylephrine [28]. Given this role, we investigated the involvement of these channels in the observed vascular changes by incubating the rings with TEA. TEA increased vascular reactivity in both groups (Figure 9A,B), with a more pronounced effect in the HCD group, as shown by the area under the curve (Figure 9C), suggesting greater potassium channel sensitivity to the refined carbohydrate-rich diet.

### 3.5. Effects of Refined Carbohydrate-Rich Diet Consumption on the Regulation of Genes Associated with Inflammation

**Figure 10:**
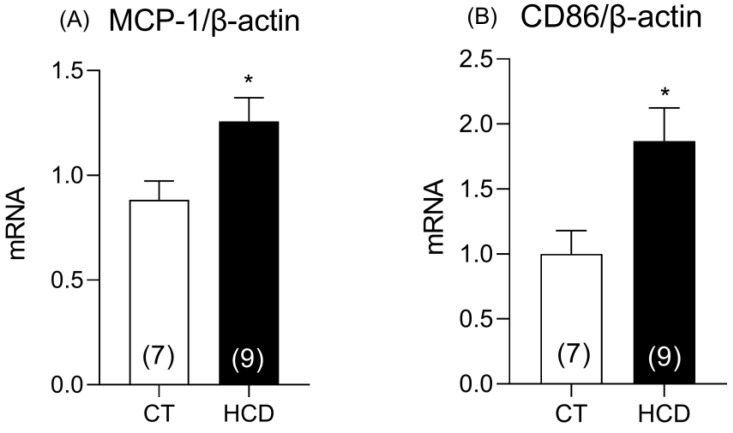
Regulation of inflammation-related genes. Concentration of messenger RNA, monocyte chemoattractant protein-1 MCP-1 (**A**), and CD86 (**B**). Data are expressed as mean ± SEM. * *p* < 0.05 vs. CT. The 95% CI = (MCP-1: 0.054 to 0.69; CD86: 0.16 to 1.57). The number of animals used is indicated in parentheses. * *p* < 0.05 HCD vs. CT. Student’s *t*-test was used.

Considering that inflammation is mediated by macrophage recruitment and infiltration, we analyzed the gene expression of MCP-1 and CD86, key components in this inflammatory process. The results showed increased expression of both genes in animals from the HCD group (Figure 10). This upregulation of MCP-1 and CD86 suggests enhanced macrophage recruitment and activation of the inflammatory response in these animals. These findings indicate that the diet may be promoting a pro-inflammatory environment, possibly contributing to endothelial dysfunction and altered vascular reactivity.

## 4. Discussion

The results demonstrate that consuming a refined carbohydrate-rich diet for 15 days reduced aortic vascular reactivity in rats. This reduction was accompanied by endothelial alterations, oxidative stress, increased NO production, and upregulation of iNOS gene expression and inflammatory markers. In addition, we observed greater involvement of potassium (K^+^) channels in vascular smooth muscle, along with elevated fasting glucose and triglyceride levels. The HCD group also showed a significant increase in the TyG index, suggesting early metabolic impairment. GTT results revealed higher glycemic levels at baseline, 15, and 30 min following glucose administration, while the ITT showed elevated glucose levels at 3 min post-insulin injection, indicating early signs of insulin resistance. However, body weight, adiposity, and total cholesterol levels remained unchanged between groups.

Under physiological conditions, the vascular endothelium plays a key role in regulating vascular function [29]. However, during endothelial dysfunction, there is an imbalance between vasodilatory and vasoconstrictive factors, accompanied by elevated levels of reactive oxygen species (ROS) and increased pro-inflammatory signaling. These changes impair vascular homeostasis and may contribute to the development of metabolic syndrome, type 2 diabetes, and cardiovascular diseases [30,31,32]. This study observed reduced vascular reactivity to phenylephrine in the aorta. Additionally, experiments conducted in endothelium-denuded rings suggest the involvement of an endothelial factor in this response.

Vascular tone regulation is largely mediated by NO, which plays a crucial role in vasodilation and cardiovascular signaling [33,34]. In addition, NO functions as a signaling molecule with well-established roles in vascular function, pathogen defense, inflammation modulation, redox homeostasis, and metabolic regulation [34].

To clarify the effects of refined carbohydrate-rich diet consumption, we incubated aortic rings with L-NAME, which resulted in increased NO release in the HCD group. We then investigated whether iNOS was involved in this increase. Vascular reactivity data and gene expression analysis indicated greater involvement of this enzyme. To further support this, we evaluated the gene expression of eNOS, which showed no difference between the groups, reinforcing the evidence that the observed increase in NO is likely associated with iNOS activity. We specifically chose to investigate iNOS rather than eNOS because, under normal conditions, NO produced by eNOS has vasodilatory effects that promote cardiovascular health by enhancing endothelial function and blood flow. However, when iNOS is induced—especially in response to chronic inflammatory stimuli—excessive NO production occurs, which can harm vascular balance. Overproduction of NO via iNOS is associated with various pathological conditions, including vascular diseases [34,35], such as atherosclerosis [36]. Thus, understanding the role of iNOS in this context is essential to elucidate the mechanisms by which refined carbohydrate-rich diets negatively affect vascular function.

Excess NO produced by iNOS can lead to the generation of reactive oxygen species, which, in turn, promote oxidative stress and damage endothelial cells. This intensified oxidative stress further exacerbates the inflammatory process, forming a self-perpetuating cycle of mutual reinforcement between inflammation and oxidative stress. This bidirectional interaction accelerates the formation of atherosclerotic plaques and impairs vascular function, establishing a vicious cycle that contributes to the progression of cardiovascular diseases [37]. Notably, incubation with Tiron only reduced phenylephrine-induced contraction in the HCD group, suggesting the involvement of vasoconstrictor free radicals.

Superoxide anion is the main free radical involved in vasoconstrictor responses within the vascular bed [38]. This radical can stimulate the expression or activity of SOD, leading, in some cases, to its dismutation into hydrogen peroxide (H_2_O_2_) [39]. Our results showed that incubation with an SOD inhibitor intensified phenylephrine-induced contraction in the HCD group, suggesting a greater role of this enzyme. Furthermore, the data also indicated increased SOD activity. The action of H_2_O_2_ in modulating vascular reactivity depends on its tissue concentration, as it can induce either relaxation or contraction. However, catalase incubation showed that H_2_O_2_ was not involved in modulating vascular reactivity under the conditions tested, as shown in Figure 8.

Several endothelium-derived factors, such as NO, promote hyperpolarization and relaxation of underlying smooth muscle cells by activating potassium (K^+^) channels [27]. Potassium channels in arterial smooth muscle cells are important modulators of vascular tone, and their dysfunction may contribute to the development of vascular diseases such as atherosclerosis [40,41]. This study showed that TEA, a potassium channel blocker, potentiated the phenylephrine-induced response in aortic segments from the HCD group. This result suggests the involvement of potassium channels in reducing vascular reactivity. In addition, it indicates that NO may contribute to the modulation of these channels, further influencing the reduced reactivity observed.

Beyond endothelial alterations, the refined carbohydrate-rich diet increased fasting glucose and triglyceride levels, in line with previous findings [42,43,44]. Hyperglycemia can contribute to elevated triglyceride levels [38,39,40] which may enhance platelet activation and promote interactions between the endothelium and lipoproteins. This interaction recruits macrophages and other pro-inflammatory cells, inducing endothelial dysfunction and vascular inflammation [42,45]. Consequently, the increased levels of blood glucose and triglycerides may raise the risk of vascular diseases [42,45,46].

In addition to these biochemical changes, the metabolic assessments provided deeper insight into the systemic effects of the refined carbohydrate-rich diet. The results of the glucose and insulin tolerance tests (GTT and ITT) revealed an enhanced glycemic response in animals exposed to the HCD. Elevated glucose levels were observed at baseline (time zero) and at 15 and 30 min after glucose injection during the GTT. Moreover, during the ITT, increased glycemic values were noted as early as 3 min after insulin administration. These findings suggest an early impairment in glucose tolerance due to insulin resistance in the HCD group [47].

Supporting these findings, the TyG index is a simple, cost-effective, and reliable indicator of insulin resistance. This surrogate marker has also been proposed as a predictor of metabolic diseases and CVD [23,48]. Analysis of the TyG index showed a significant increase in the HCD group compared to the CT group, indicating early signs of metabolic impairment. Such metabolic disturbances may further exacerbate endothelial dysfunction and inflammatory signaling, potentially accelerating the pathogenesis of cardiovascular and metabolic diseases [49].

Despite these marked metabolic changes, the refined carbohydrate-rich diet did not induce weight gain or increased adiposity in the animals. This finding contrasts with previous studies that reported the development of obesity in female rats subjected to the same diet for 15 days [16,17]. Such a discrepancy suggests that the response to refined carbohydrate intake may be modulated by sex-specific biological and hormonal factors, which we intend to explore in future investigations. Current evidence indicates that estrogens—either through direct action or via activation of their receptors in adipocytes and adipose tissue—play a central role in adipose tissue deposition and regulation [50,51].

Thus, the results of this study reinforce the strong association between refined carbohydrate intake and the development of insulin resistance [52]. Furthermore, they support the idea of a link between insulin resistance and cardiovascular dysfunction [53].

Cardiovascular diseases have been widely associated with chronic low-grade inflammation, with macrophages as key players in this process [53]. Monocyte chemoattractant protein1 (MCP-1) is one of the main chemokines that regulates macrophage migration and infiltration [54]. It also modulates the expression of other inflammatory factors, promoting a complex network of cellular interactions that perpetuate inflammation and contribute to endothelial dysfunction [55]. In this study, gene expression results showed increased levels of this marker.

The role of MCP-1 in atheroma plaque formation is well established. This chemokine attracts circulating monocytes to the vascular endothelium, where they differentiate into macrophages [55,56]. Through scavenger receptors, these macrophages take up oxidized LDL and give rise to foam cells. Foam cells, in turn, release inflammatory cytokines, enzymes, and growth factors that, along with cytokines from activated T cells, amplify the inflammatory response and drive the formation of atherosclerotic plaques [55,56].

In addition to MCP-1, the results also demonstrated increased expression of CD86. CD86 expression is markedly elevated in intermediate and non-classical monocytes, which are highly pro-inflammatory and contribute to the development of atherosclerosis [57]. Furthermore, the number of CD86+ B cells correlates with increased stenosis and a higher incidence of stroke in humans [58].

This study demonstrated that the consumption of a refined carbohydrate-rich diet for 15 days increased blood glucose levels, which in turn may contribute to elevated triglycerides. These changes may promote interactions between the endothelium and lipoproteins, facilitating macrophage recruitment via markers such as MCP-1 and CD86. Additionally, iNOS expression is upregulated by inflammatory stimuli, particularly pro-inflammatory cytokines. Increased activity of this enzyme leads to greater NO production, which, in this study, resulted in reduced vascular reactivity. Additionally, a significant increase in the TyG index, as well as elevated glycemic levels during the GTT and ITT, further supports the metabolic dysregulation associated with refined carbohydrate intake. Together, these factors contribute to the development of oxidative stress, creating a vicious cycle between oxidative stress and inflammation.

This study is limited by the exclusive use of male animals, which was intentional to avoid the interference of cyclical hormonal fluctuations present in females that could impact metabolic and vascular analyses and complicate the interpretation of the results. However, we recognize that dietary effects may differ between sexes, and therefore, future studies involving females are necessary to investigate these potential differences.

## 5. Conclusions

**Figure 11:**
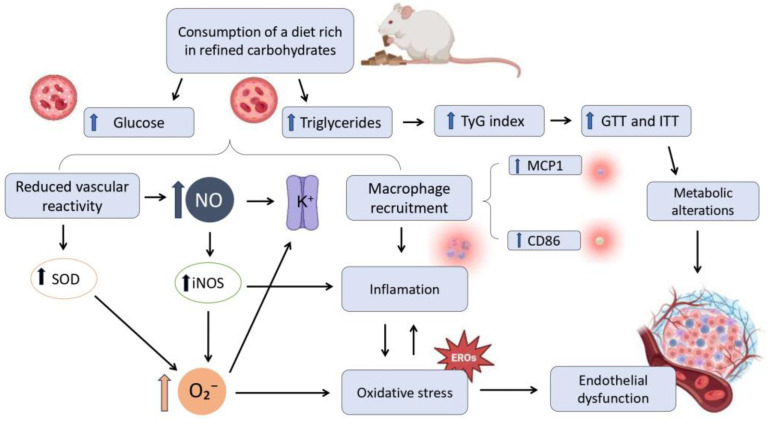
Schematic representation of the proposed mechanisms by which short-term consumption of a refined carbohydrate-rich diet impairs vascular function and metabolism. The consumption of a refined carbohydrate-rich diet leads to increased circulating levels of glucose and triglycerides. This increase is associated with a rise in the TyG index (triglycerides and glucose), a marker widely used to estimate insulin resistance. Consequently, there is a worsening in glucose tolerance (GTT) and insulin tolerance (ITT) tests, indicating the presence of significant metabolic alterations that contribute to endothelial dysfunction.

In summary, the consumption of a refined carbohydrate-rich diet for only 15 days was sufficient to induce vascular alterations, increased production of nitric oxide (NO) and reactive oxygen species, and activation of inflammatory pathways in rats. These findings highlight the early impact of this dietary pattern on endothelial dysfunction and emphasize the importance of preventive strategies against excessive consumption of simple sugars. The refined carbohydrate-rich diet impaired vascular reactivity independently of weight gain, which was only observed in male rats during the study. This association between refined carbohydrate intake, inflammation, and oxidative stress suggests that even short-term consumption can promote significant endothelial changes, acting as risk factors for the development of cardiovascular diseases. Furthermore, these results contribute to elucidating the underlying mechanisms and may aid in identifying potential therapeutic targets. However, further studies, including female animals, are needed to better understand whether sex-specific differences influence these outcomes and to fully clarify the involved mechanisms. A summary of the main experimental findings is schematically presented in Figure 11, illustrating the effects of consuming a diet rich in refined carbohydrates on metabolic parameters, inflammation, oxidative stress, and vascular function.

From a vascular perspective, chronic exposure to this metabolic profile leads to reduced vascular reactivity. The increase in the activity of the antioxidant enzyme superoxide dismutase may be related to the increase in the production of superoxide anion (O_2_^−^), acting in an attempt to neutralize this radical. In parallel, an increase in nitric oxide (NO) production is observed, mediated by the upregulation of the inducible nitric oxide synthase (iNOS) enzyme.

However, excess NO in a pro-oxidant environment favors its interaction with O_2_^−^, generating even more toxic reactive species, which further exacerbate oxidative stress. Additionally, factors such as NO and O_2_^−^ promote the hyperpolarization and relaxation of vascular smooth muscle cells through the activation of potassium (K^+^) channels, thereby favoring vasorelaxation and consequently contributing to the reduced vascular reactivity observed. This pro-inflammatory and pro-oxidant environment also promotes macrophage recruitment to tissues, characterized by the increased expression of inflammatory markers such as MCP-1 (monocyte chemoattractant protein-1) and CD86 (a macrophage activation marker). The activation of these immune cells contributes to the maintenance of a chronic inflammatory state. Inflammation, combined with persistent oxidative stress, establishes a feedback loop that further exacerbates cellular and vascular damage. As a result, these alterations culminate in endothelial dysfunction, a key event in the development of cardiovascular diseases, in addition to worsening the metabolic disturbances associated with excessive refined carbohydrate consumption.

## Figures and Tables

**Table 1:** Body weight, food intake, water intake, and energy efficiency.

	CT	HCD
Initial body weight (g)	367 ± 39	354 ± 27
Final body weight (g)	418 ± 43	405 ± 30
Weigh gain (g)	50 ± 3.1	50 ± 2.7
Food intake (g/day)	27 ± 1.2	20 ± 0.5 *
Water intake (mL/day)	39 ± 6.3	23 ± 3.4 *
Energy efficiency (Kcal)	0.45 ± 0.07	0.54 ± 0.07 *

Data are expressed as mean ± SEM (*n* = 8). * *p* < 0.05 HCD vs. CT. Student’s *t*-test was used.

**Table 2:** Effects of refined carbohydrate-rich diet consumption on body parameters.

Adipose Tissue	CT	HCD
Epididymal (g)	12 ± 1.01	11.72 ± 1.16
Mesenteric (g)	4.41 ± 0.38	4.35 ± 0.32
Subcutaneous (g)	12.22 ± 0.75	11.8 ± 0.54
Perirenal (g)	1.98 ± 0.17	1.68 ± 0.13
Retroperitoneal (g)	8.93 ± 0.56	9.38 ± 0.67
Thoracic PVAT (g)	0.26 ± 0.012	0.22 ± 0.013

Data are expressed as mean ± SEM (*n* = 8).

**Table 3:** Effects of refined carbohydrate-rich diet consumption on glycemic and lipid parameters.

Adipose Tissue	CT	HCD
Glucose (mg/dL)	117.5 ± 5.3	130.3 ± 11.1 *
Triglyceride (mg/dL)	105 ± 23.8	197.04 ± 29.1 *
Total Cholesterol (mg/dL)	110.4 ± 17.4	126.8 ± 9.0
HDL (mg/dL)	141.65 ± 10.5	144.12 ± 15.5
TyG index	12.4 ± 2.9	25.4 ± 4.5 *

Data are expressed as mean ± SEM (*n* = 6–8). * *p* < 0.05 HCD vs. CT. Student’s *t*-test was used.

## Data Availability

The original contributions presented in this study are included in the article. Further inquiries can be directed to the corresponding author(s).

## Abbreviations

The following abbreviations are used in this manuscript:

CD86	Cluster of differentiation 86.
CT	Control.
CVDs	Cardiovascular diseases.
DETCA	Diethyldithiocarbamic acid.
eNOS	Endothelial nitric oxide synthase.
GTT	Glucose tolerance test.
HCD	High-carbohydrate diet.
iNOS	Inducible nitric oxide synthase.
ITT	Insulin tolerance test.
mRNA	Messenger ribonucleic acid.
NO	Nitric oxide.
NOS	Nitric oxide synthase.
PCR	Polymerase chain reaction.
PVAT	Perivascular adipose tissue.
qPCR	Quantitative polymerase chain reaction.
RNA	Ribonucleic acid.
ROS	Reactive oxygen species.
SOD	Superoxide dismutase.
TyG	Triglyceride–glucose index.

## References

[1] Arnone D., Chabot C., Heba A.-C., Kökten T., Caron B., Hansmannel F., Dreumont N., Ananthakrishnan A.N., Quilliot D., Peyrin-Biroulet L. (2022). Sugars and Gastrointestinal Health. Clin. Gastroenterol. Hepatol..

[2] Azais-Braesco V., Sluik D., Maillot M., Kok F., Moreno L.A. (2017). A review of total & added sugar intakes and dietary sources in Europe. Nutr. J..

[3] Witek K., Wydra K., Filip M. (2022). A high-sugar diet consumption, metabolism and health impacts with a focus on the development of substance use disorder: A narrative review. Nutrients.

[4] World Health Organization Cardiovascular Diseases (CVDs). https://www.who.int/news-room/fact-sheets/detail/cardiovascular-diseases-(cvds).

[5] Zhang Y., Giovannucci E.L. (2022). Ultra-processed foods and health: A comprehensive review. Crit. Rev. Food Sci. Nutr..

[6] Dai S., Wellens J., Yang N., Li D., Wang J., Wang L., Yuan S., He Y., Song P., Munger R. (2024). Ultra-processed foods and human health: An umbrella review and updated meta-analyses of observational evidence. Clin. Nutr..

[7] Lv J.-L., Wei Y.-F., Sun J.-N., Shi Y.-C., Liu F.-H., Sun M.-H., Chang Q., Wu Q.-J., Zhao Y.-H. (2024). Ultra-processed food consumption and metabolic disease risk: An umbrella review of systematic reviews with meta-analyses of observational studies. Front. Nutr..

[8] Yang Q., Zhang Z., Gregg E.W., Flanders W.D., Merritt R., Hu F.B. (2014). Added sugar intake and cardiovascular diseases mortality among US adults. JAMA Intern. Med..

[9] Dehghan M., Mente A., Zhang X., Swaminathan S., Li W., Mohan V., Iqbal R., Kumar R., Wentzel-Viljoen E., Rosengren A. (2017). Associations of fats and carbohydrate intake with cardiovascular disease and mortality in 18 countries from five continents (PURE): A prospective cohort study. Lancet.

[10] Mohammadifard N., Mansourian M., Firouzi S., Taheri M., Haghighatdoost F. (2021). Longitudinal association of dietary carbohydrate and the risk cardiovascular disease: A dose-response meta-analysis. Crit. Rev. Food Sci. Nutr..

[11] Shi Z., Zhu W., Lei Z., Yan X., Zhang X., Wei S., Wang Q. (2024). Intake of added sugar from different sources and risk of all-cause mortality and cardiovascular diseases: The role of body mass index. J. Nutr..

[12] Jo U., Park K. (2023). Carbohydrate intake and risk of cardiovascular disease: A systematic review and meta-analysis of prospective studies. Nutrients.

[13] Huang C., Liang Z., Ma J., Hu D., Yao F., Qin P. (2023). Total sugar, added sugar, fructose, and sucrose intake and all-cause, cardiovascular, and cancer mortality: A systematic review and dose-response meta-analysis of prospective cohort studies. Nutrition.

[14] World Health Organization (2015). Guideline: Sugars Intake for Adults and Children [Internet].

[15] Ferreira A.V.M., Mario É.G., Porto L.C.J., Andrade S.P., Botion L.M. (2010). High-carbohydrate diet selectively induces tumor necrosis factor-α production in mice liver. Inflammation.

[16] Niño O.M., da Costa C.S., Torres K.M., Zanol J.F., Freitas-Lima L.C., Miranda-Alves L., Graceli J.B. (2020). High-refined carbohydrate diet leads to polycystic ovary syndrome-like features and reduced ovarian reserve in female rats. Toxicol. Lett..

[17] Zanol J.F., Niño O.M., da Costa C.S., Zimerman J., Silva N.P., Oliveira T.M., Maas E.M., dos Santos F.C., Miranda-Alves L., Graceli J.B. (2022). High-refined carbohydrate diet alters different metabolic functions in female rats. Mol. Cell. Endocrinol..

[18] Oliveira M.C., Menezes-Garcia Z., Henriques M.C.C., Soriani F.M., Pinho V., Faria A.M.C., Santiago A.F., Cara D.C., Souza D.G., Teixeira M.M. (2012). Acute and sustained inflammation and metabolic dysfunction induced by high refined carbohydrate-containing diet in mice. Obesity.

[19] dos Reis Costa D.E.F., Silveira A.L.M., Campos G.P., Nóbrega N.R.C., De Araújo N.F., de Figueiredo Borges L., dos Santos Aggum Capettini L., Ferreira A.V.M., Bonaventura D. (2021). High-carbohydrate diet enhanced the anticontractile effect of perivascular adipose tissue through activation of renin-angiotensin system. Front. Physiol..

[20] Bruno A.S., Castor R.G.M., Berg B., Costa D.E.F.d.R., Monteiro A.L.L., Scalzo S., Oliveira K.C.M., Bello F.L.M., Aguiar G.C., Melo M.B. (2023). Cardiac disturbances and changes in tissue cytokine levels in mice fed with a high-refined carbohydrate diet. Cytokine.

[21] Higashi Y. (2022). Roles of oxidative stress and inflammation in vascular endothelial dysfunction-related disease. Antioxidants.

[22] Masi L.N., Martins A.R., Crisma A.R., do Amaral C.L., Davanso M.R., Serdan T.D.A., da Cunha de Sá R.D.C., Cruz M.M., Alonso-Vale M.I.C., Torres R.P. (2017). Combination of a high-fat diet with sweetened condensed milk exacerbates inflammation and insulin resistance induced by each separately in mice. Sci. Rep..

[23] Simental-Mendía L.E., Guerrero-Romero F. (2020). The correct formula for the triglycerides and glucose index. Eur. J. Pediatr..

[24] Nunes K.Z., Fioresi M., Marques V.B., Vassallo D.V. (2018). Acute copper overload induces vascular dysfunction in aortic rings due to endothelial oxidative stress and increased nitric oxide production. J. Toxicol. Environ. Health Part A.

[25] Bolsoni-Lopes A., Festuccia W.T., Farias T.S.M., Chimin P., Torres-Leal F.L., Derogis P.B.M., de Andrade P.B., Miyamoto S., Lima F.B., Curi R. (2013). Palmitoleic acid (n-7) increases white adipocyte lipolysis and lipase content in a PPARα-dependent manner. Am. J. Physiol. Metab..

[26] Misra H.P., Fridovich I. (1972). The role of superoxide anion in the autoxidation of epinephrine and a simple assay for superoxide dismutase. J. Biol. Chem..

[27] Félétou M. (2009). Calcium-activated potassium channels and endothelial dysfunction: Therapeutic options?. Br. J. Pharmacol..

[28] Nelson M.T., Quayle J.M. (1995). Physiological roles and properties of potassium channels in arterial smooth muscle. Am. J. Physiol..

[29] Xu S., Ilyas I., Little P.J., Li H., Kamato D., Zheng X., Luo S., Li Z., Liu P., Han J. (2021). Endothelial dysfunction in atherosclerotic cardiovascular diseases and beyond: From mechanism to pharmacotherapies. Pharmacol. Rev..

[30] Sun H.-J., Wu Z.-Y., Nie X.-W., Bian J.-S. (2020). Role of endothelial dysfunction in cardiovascular diseases: The link between inflammation and hydrogen sulfide. Front. Pharmacol..

[31] Allbritton-King J.D., García-Cardeña G. (2023). Endothelial cell dysfunction in cardiac disease: Driver or consequence?. Front. Cell Dev. Biol..

[32] Ray A., Maharana K.C., Meenakshi S., Singh S. (2023). Endothelial dysfunction and its relation in different disorders: Recent update. Heal. Sci. Rev..

[33] Förstermann U., Nakane M., Tracey W.R., Pollock J.S. (1993). Isoforms of nitric oxide synthase: Functions in the cardiovascular system. Eur. Heart J..

[34] Roy R., Wilcox J., Webb A.J., O’gallagher K. (2023). Dysfunctional and dysregulated nitric oxide synthases in cardiovascular disease: Mechanisms and therapeutic potential. Int. J. Mol. Sci..

[35] Cinelli M.A., Do H.T., Miley G.P., Silverman R.B. (2019). Inducible nitric oxide synthase: Regulation, structure, and inhibition. Med. Res. Rev..

[36] Lind M., Hayes A., Caprnda M., Petrovic D., Rodrigo L., Kruzliak P., Zulli A. (2017). Inducible nitric oxide synthase: Good or bad?. Biomed. Pharmacother..

[37] Zhang Z., Guo J. (2024). Deciphering Oxidative stress in cardiovascular disease progression: A blueprint for mechanistic understanding and therapeutic innovation. Antioxidants.

[38] Wattanapitayakul S.K., Bauer J.A. (2001). Oxidative pathways in cardiovascular disease: Roles, mechanisms, and therapeutic implications. Pharmacol. Ther..

[39] Wang Y., Branicky R., Noë A., Hekimi S. (2018). Superoxide dismutases: Dual roles in controlling ROS damage and regulating ROS signaling. J. Cell Biol..

[40] Sahranavard T., Carbone F., Montecucco F., Xu S., Al-Rasadi K., Jamialahmadi T., Sahebkar A. (2020). The role of potassium in atherosclerosis. Eur. J. Clin. Investig..

[41] Lemke J., Gollasch M., Tsvetkov D., Schulig L. (2025). Advances in the design and development of chemical modulators of the voltage-gated potassium channels K _V_ 7.4 and K _V_ 7.5. Expert Opin. Drug Discov..

[42] DiNicolantonio J.J., Lucan S.C., O’kEefe J.H. (2016). The evidence for saturated fat and for sugar related to coronary heart disease. Prog. Cardiovasc. Dis..

[43] Spadaro P.A., Naug H.L., DU Toit E.F., Donner D., Colson N.J. (2015). A refined high carbohydrate diet is associated with changes in the serotonin pathway and visceral obesity. Genet. Res..

[44] Te Morenga L.A., Howatson A.J., Jones R.M., Mann J. (2014). Dietary sugars and cardiometabolic risk: Systematic review and meta-analyses of randomized controlled trials of the effects on blood pressure and lipids. Am. J. Clin. Nutr..

[45] Bradley P. (2019). Refined carbohydrates, phenotypic plasticity and the obesity epidemic. Med. Hypotheses.

[46] Ma X., Nan F., Liang H., Shu P., Fan X., Song X., Hou Y., Zhang D. (2022). Excessive intake of sugar: An accomplice of inflammation. Front. Immunol..

[47] Zhan X., Wang L., Wang Z., Chai S., Zhu X., Ren W., Chang X. (2019). High-glucose administration induces glucose intolerance in mice: A critical role of toll-like receptor 4. J. Clin. Biochem. Nutr..

[48] Gounden V., Devaraj S., Jialal I. (2024). The role of the triglyceride-glucose index as a biomarker of cardio-metabolic syndromes. Lipids Heal. Dis..

[49] Sun Y., Rawish E., Nording H.M., Langer H.F. (2021). Inflammation in metabolic and cardiovascular disorders—Role of oxidative stress. Life.

[50] Palmer B.F., Clegg D.J. (2015). The sexual dimorphism of obesity. Mol. Cell Endocrinol..

[51] Kuryłowicz A. (2023). Estrogens in adipose tissue physiology and obesity-related dysfunction. Biomedicines.

[52] Barazzoni R., Deutz N.E.P., Biolo G., Bischoff S., Boirie Y., Cederholm T., Cuerda C., Delzenne N., Leon Sanz M., Ljungqvist O. (2017). Carbohydrates and insulin resistance in clinical nutrition: Recommendations from the ESPEN expert group. Clin. Nutr..

[53] Kosmas C.E., Bousvarou M.D., Kostara C.E., Papakonstantinou E.J., Salamou E., Guzman E. (2023). Insulin resistance and cardiovascular disease. J. Int. Med. Res..

[54] Deshmane S.L., Kremlev S., Amini S., Sawaya B.E. (2009). Monocyte chemoattractant Protein-1 (MCP-1): An overview. J. Interf. Cytokine Res..

[55] Singh S., Anshita D., Ravichandiran V. (2021). MCP-1: Function, regulation, and involvement in disease. Int. Immunopharmacol..

[56] Ramji D.P., Davies T.S. (2015). Cytokines in atherosclerosis: Key players in all stages of disease and promising therapeutic targets. Cytokine Growth Factor. Rev..

[57] Liu F., Wang Y., Yu J. (2023). Role of inflammation and immune response in atherosclerosis: Mechanisms, modulations, and therapeutic targets. Hum. Immunol..

[58] Mantani P.T., Ljungcrantz I., Andersson L., Alm R., Hedblad B., Björkbacka H., Nilsson J., Fredrikson G.N. (2014). Circulating CD40^+^ and CD86^+^ B cell subsets demonstrate opposing associations with risk of stroke. Arter. Thromb. Vasc. Biol..

